# Enhancing Energy Harvesting Efficiency of Flapping Wings with Leading-Edge Magnus Effect Cylinder

**DOI:** 10.3390/biomimetics9050293

**Published:** 2024-05-13

**Authors:** Huaqiang Zhang, Bing Zhu, Weidong Chen

**Affiliations:** 1 School of Energy and Power Engineering, University of Shanghai for Science and Technology, Shanghai 200093, China; z1453299046@163.com; 2 National Key Laboratory of Marine Engine Science and Technology, Shanghai 201108, China; 3 Jiangsu Kingkind Industrial Furnace Co., Ltd., Yancheng 224100, China; chenqianwd@163.com

**Keywords:** flapping wing, energy harvesting, Magnus effect, leading-edge vortex, trailing-edge vortex

## Abstract

According to the Magnus principle, a rotating cylinder experiences a lateral force perpendicular to the incoming flow direction. This phenomenon can be harnessed to boost the lift of an airfoil by positioning a rotating cylinder at the leading edge. In this study, we simulate flapping-wing motion using the sliding mesh technique in a heaving coordinate system to investigate the energy harvesting capabilities of Magnus effect flapping wings (MEFWs) featuring a leading-edge rotating cylinder. Through analysis of the flow field vortex structure and pressure distribution, we explore how control parameters such as gap width, rotational speed ratio, and phase difference of the leading-edge rotating cylinder impact the energy harvesting characteristics of the flapping wing. The results demonstrate that MEFWs effectively mitigate the formation of leading-edge vortices during wing motion. Consequently, this enhances both lift generation and energy harvesting capability. MEFWs with smaller gap widths are less prone to induce the detachment of leading-edge vortices during motion, ensuring a higher peak lift force and an increase in the energy harvesting efficiency. Moreover, higher rotational speed ratios and phase differences, synchronized with wing motion, can prevent leading-edge vortex generation during wing motion. All three control parameters contribute to enhancing the energy harvesting capability of MEFWs within a certain range. At the examined Reynolds number, the optimal parameter values are determined to be $a^{*}$ = 0.0005, *R* = 3, and $\phi_{0}$ = 0°.

## 1. Introduction

Drawing inspiration from avian, insectoid, and marine fauna, McKinney and Delaurier pioneered a novel approach to energy harvesting. Specifically, they proposed the utilization of flapping-wing mechanisms to extract energy from fluid mediums [1]. In contrast to conventional rotating-blade-based energy harvesting apparatus, flapping-wing energy harvesting devices offer several advantages. These include a simpler design, reduced noise output, sustained high operational efficiency even in shallow or low-speed environments, and adaptability to a variety of usage scenarios [2,3,4]. Therefore, flapping-wing energy harvesting devices hold immense promise for further development and widespread application.

Presently, numerous researchers have delved into the investigation of flapping-wing energy harvesting systems, primarily focusing on several key aspects. These include the geometric parameters of flapping wings, such as thickness and chord length [5]; motion parameters, encompassing the heave amplitude [6], pitch amplitude [7], phase difference between heave and pitch [8], motion frequency [9], and trajectory [10]; operational parameters like the Reynolds number (Re) [11]; and the effects of constraining environments, such as free surfaces [12] and ground effects [13]. Moreover, researchers have explored various active and passive control methodologies, such as flexible deformation [14,15], active flaps [16], and oscillating tails [17], aimed at enhancing the lift of flapping wings and thereby augmenting energy harvesting efficiency.

Based on the Magnus effect, the amalgamation of a rotating cylinder with an airfoil presents a promising avenue of research. As early as 1924, Reid and Flettner [18] independently explored this composite wing for lift augmentation and drag reduction. Initial investigations primarily relied on experimental methodologies, with limited exploration of underlying mechanisms. With the advancement in simulation technology, this research has evolved into a more systematic and mature field. Modi [19] investigated the integration of Magnus effect cylinders into symmetrical airfoils of varying configurations, assessing the impact of cylinder embedding at different positions on overall airfoil performance. At high angles of attack (α ≈ 30°), the lift coefficient saw an increase of over 200%. Ahmed et al. [20] conducted numerical analyses on an NACA0024 airfoil embedded with a rotating cylinder, observing a 36% rise in the lift coefficient compared to the original airfoil and a 122% delay in the stall angle. Huda et al. [21] numerically analyzed a front-edge cylinder of an NACA0010 airfoil at different rotation speeds, demonstrating a maximum lift increase of 145%. Notably, the front-edge cylinder not only enhanced lift at the airfoil’s leading edge but also ameliorated flow separation at the rear. Ali et al. [22] embedded rotating cylinders in S1223 airfoils and flat plates, conducting numerical simulations under varied conditions, including rotation speeds ranging from 500 RPM to 1000 RPM, inflow velocities from 5 m/s to 30 m/s, and angles of attack from 0 to 20 degrees. The lift coefficients of the improved airfoil and flat plate increased by 39% and 128%, respectively. In 2012, Seifert [23] provided a comprehensive literature review and prospects regarding the application of the Magnus effect in aerospace, serving as a valuable reference for researchers in this domain. Overall, embedding rotating cylinders at the leading edge of an airfoil indeed significantly enhances the lift coefficient.

The ratio of cylinder rotation speed to incoming flow velocity (i.e., rotation speed ratio) is an important control parameter affecting the lift of Magnus effect airfoils. Mokhtarian et al. numerically analyzed the effects of leading-edge rotating cylinders on symmetrical and asymmetrical airfoils [24,25], finding that at rotation speed ratios of 1 and 2, both types of airfoils experienced increased lift and stall angles. Tennant et al. recorded changes in the lift coefficient [26] and boundary layer [27] for airfoils with trailing-edge rotating cylinders at rotation speed ratios of 3 and 4 and summarized the current applications of the Magnus effect [28], finding that the trends in lift curve changes at high and low rotation speed ratios were opposite. Al-Garni conducted experimental studies on an NACA0024 airfoil with leading-edge rotating cylinders and flaps [20], finding that resistance increased with increasing rotation speed ratio. Additionally, the gap between the rotating cylinder and the airfoil is a key parameter affecting aerodynamic performance. Abdulla et al. [29] determined, through numerical simulations at rotation ratios of 1, 2, and 3, that maintaining a gap of 3 mm resulted in the maximum lift-to-drag ratio. Najdat [30] analyzed the effects of different rotation speed ratios, angles of attack, and gaps on the aerodynamic performance of trailing-edge rotating cylinders through numerical and experimental methods.

As evidenced by numerous studies on fixed airfoils, harnessing the Magnus effect of rotating cylinders has demonstrated the potential to increase the lift of stationary airfoils at fixed angles of attack. The rotation speed ratio and the gap between the leading-edge cylinder are identified as crucial control parameters affecting the lift enhancement in airfoils. However, existing research has predominantly focused on static airfoils, showcasing that embedding Magnus cylinders enhances the lift of airfoils at zero and low angles of attack. Yet, there remains a gap in research regarding the application of the Magnus effect to oscillating wings in energy harvesting devices. Given the time-varying angle of attack of oscillating wings, it remains uncertain whether the conclusions drawn from static airfoil Magnus research are applicable. This paper proposes, for the first time, the application of a Magnus wing with a leading-edge-embedded rotating cylinder to a flapping-wing energy harvesting device, aiming to enhance the device’s energy harvesting efficiency. Employing the sliding mesh technique in a relative heaving coordinate system, numerical methods are utilized to investigate the energy harvesting characteristics of a leading-edge Magnus flapping wing. This exploration delves into the underlying mechanisms driving changes in energy harvesting efficiency caused by the Magnus effect in flapping wings and aims to identify optimized parameter ranges for the gap, rotation speed ratio, and motion phase difference. This study thus offers valuable insights for the design of Magnus flapping-wing energy harvesting devices.

## 2. Numerical Model

### 2.1. Physical Model and Numerical Methods

**Figure 1:**
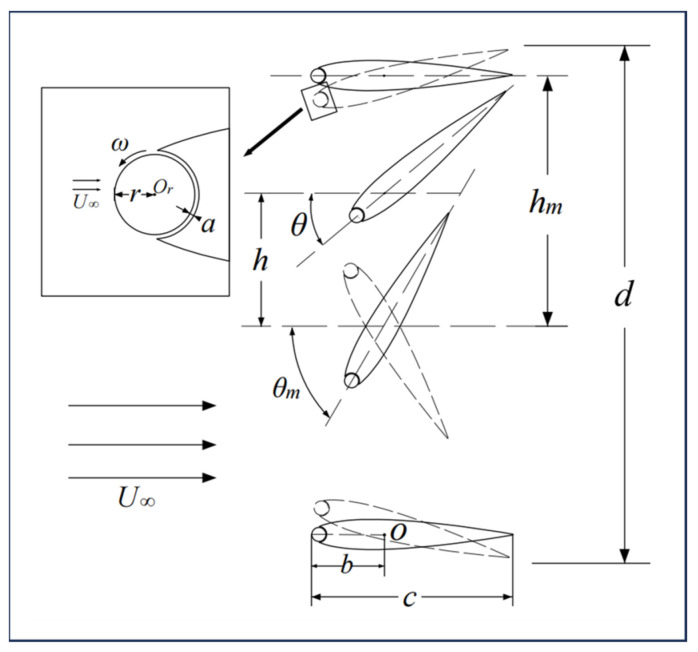
Schematic diagram of MEFW motion and parameter annotations.

Figure 1 illustrates the motion schematic of the Magnus effect flapping-wing (MEFW) device, where the airfoil with a leading-edge rotating cylinder is referred to as the MEFW throughout this text. Based on the NACA0015 foil, a circle with a radius of *r* is embedded within the leading edge of the airfoil, while maintaining the chord length *c* constant. The gap between the circle and the main wing is denoted as *a*. Assuming a free stream velocity of $U_{\infty }$, the pitching axis is located at a distance *b* = *c*/3 from the leading edge of the airfoil, and the pitching angle is denoted as *θ*. The pitching center *O* undergoes an up-and-down displacement *h* in the *y* direction. The motion of the MEFW involves a combination of pitching and heaving motion, which can be described as

(1) $$\theta =\theta_{m}\sin(2\pi ft),$$

(2) $$h=h_{m}\sin\left(2\pi ft+\phi \right),$$

where $\theta_{m}$ represents the given maximum pitching amplitude, *f* denotes the pitching frequency, *t* indicates time, and ϕ is the phase angle difference between heaving and pitching motions, with a value of 90°. To ensure that the motion of the leading-edge rotating cylinder is synchronized with the overall motion of the flap, it is assumed that the leading-edge rotating cylinder of the MEFW airfoil is controlled by a sinusoidal motion with a rotation angular speed ω around its own center $O_{r}$, described as

(3) $$\omega =\omega_{m}\sin(2\pi ft+\phi_{0}),$$

where $\omega_{m}$ is the maximum angular velocity amplitude of the cylinder rotation, and $\phi_{0}$ is the phase difference between the pitching motion of the flap and the rotation motion of the leading-edge rotating cylinder. The ratio of the angular velocity amplitude $\omega_{m}r$ to the freestream velocity $U_{\infty }$ is defined as the rotational speed ratio $R=\omega_{m}r/U_{\infty }$.

During the motion of the MEFW, the fluid exerts work on the flap primarily through the contributions of the lift force $F_{y}$ and the pitching moment *M*. The power of the lift force work $P_{y}\left(t\right)$ and the power of the pitching moment work $P_{\theta }\left(t\right)$ can be respectively expressed as

(4) $$P_{y}\left(t\right)=F_{y}(t)V_{y}(t),$$

(5) $$P_{\theta }\left(t\right)=M\left(t\right)\dot{\theta }\left(t\right).$$

The average power coefficient of lift force and moment over one pitching motion cycle can be respectively calculated as

(6) $${\bar{C}}_{ph}=\int_{0}^{1}\left(C_{y}\left(t\right)\frac{V_{y}\left(t\right)}{U_{\infty }}\right)d\left(t/T\right),$$

(7) $${\bar{C}}_{p\theta }=\int_{0}^{1}\left(C_{m}\left(t\right)\frac{\dot{\theta }\left(t\right)c}{U_{\infty }}\right)d\left(t/T\right),$$

where $V_{y}$ and $\dot{\theta }$ represent the heave velocity and the pitching angular velocity, respectively. *T =* 1/*f* denotes the pitching motion period. $C_{y}$ and $C_{m}$ denote the lift coefficient and the moment coefficient, respectively, defined as $C_{y}=\frac{F_{y}}{0.5\rho c^{2}U_{\infty }^{2}}$ and $C_{m}=\frac{M}{0.5\rho c^{3}U_{\infty }^{2}}$.

Additionally, when calculating the efficiency of the MEFW system, the influence of power consumed by driving the rotation of the leading-edge cylinder needs to be considered. The coefficient of the torque required for the cylinder rotation, denoted as ${\bar{C}}_{p\omega }$, is defined as

(8) $${\bar{C}}_{p\omega }=\int_{0}^{1}\left(C_{m\omega }\left(t\right)\frac{\omega \left(t\right)c}{U_{\infty }}\right)d\left(t/T\right).$$

Here, $C_{m\omega }$ represents the moment coefficient of the rotating cylinder, which will be explained in detail in the following text. The total power coefficient of the MEFW, denoted as ${\bar{C}}_{p}$, is the sum of the overall lift power coefficient, the pitch moment power coefficient, and the torque power coefficient of the rotating cylinder (negative value), defined as

(9) $${\bar{C}}_{p}={\bar{C}}_{ph}+{\bar{C}}_{p\theta }+{\bar{C}}_{p\omega }.$$

When ${\bar{C}}_{p}>0$, the fluid performs positive work on the MEFW, indicating that the wing extracts energy from the fluid. Conversely, when the fluid performs negative work on the wing, additional energy is required for the flapping motion. The efficiency η of the MEFW represents the ratio of energy obtained during one motion cycle to the energy of the incoming flow, expressed as

(10) $$\eta ={\bar{C}}_{p}\frac{c}{d},$$

where *d* represents the maximum displacement of the MEFW in the vertical direction.

### 2.2. Computational Method and Domain Discretization

**Figure 2:**
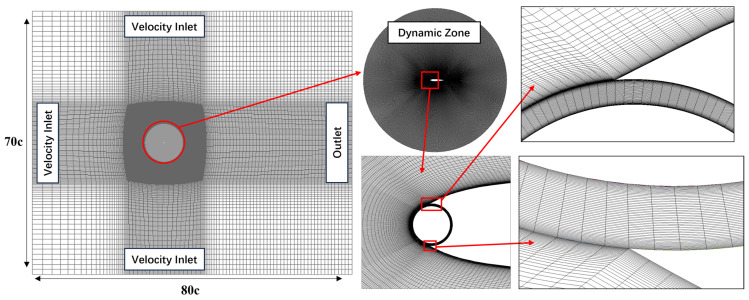
Computational domain and grid distribution.

The computational domain and grid distribution used in this study are illustrated in Figure 2. The overall size of the computational domain is 80c × 70c. To simplify the handling of large-amplitude motions, reduce the convergence time of simulation results, and ensure the stability of the numerical computation process, this study investigates the wing motion in a relative heaving coordinate system. The basic idea proposed by Kinsey and Dumas [7] is to add acceleration source terms to the momentum equation and couple the time-varying heave velocity on the boundary, thereby only examining the pitching motion of the wing in the heaving coordinate system. The upstream, top, and bottom of the computational domain are imposed with transient velocity boundary conditions using User-Defined Functions (UDFs), and the rotation of the cylinder is controlled in real time through the program to adjust the rotation center and pitch angular velocity. The downstream outlet is assigned with static pressure boundary conditions. A sliding mesh interface is used to connect and exchange data between the pitching motion region and the stationary region, while the wing surface is subjected to a no-slip wall boundary condition.

The Reynolds-averaged Navier–Stokes equations are solved using a pressure–velocity coupling algorithm, and the Spalart–Allmaras (S-A) turbulence model, commonly used for external flow simulations, is employed. In the discretization of equations, a second-order accuracy scheme is adopted for all spatial and temporal terms, and numerical computations are performed in double precision.

**Figure 3:**
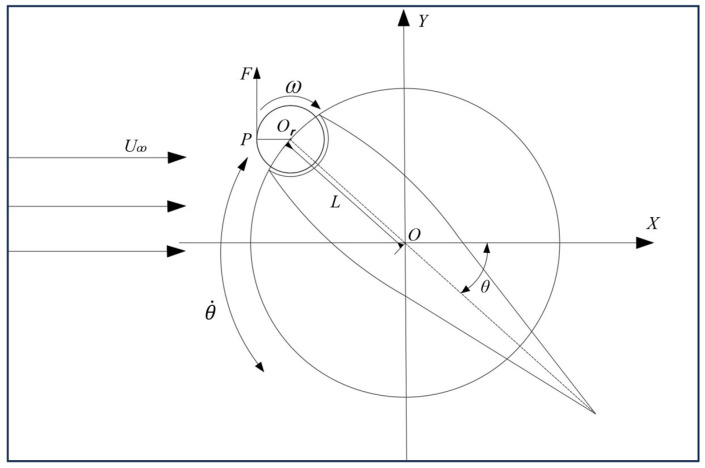
Schematic diagram illustrating the application of the shifting axis theorem for the cylinder.

In the relative heaving coordinate system, the torque of the rotating cylinder monitored in this numerical simulation is referenced to the coordinate origin *O*. Therefore, for the final efficiency calculation, the monitored torque values need to be transformed into torque values relative to the center ($O_{r}$) of the cylinder using the theorem of shifting axis [31]. Figure 3 depicts a schematic diagram of the MEFW at a certain instant.

Assuming the force acting on any point *P* on the rotating cylinder is *F*, the torque at point *P* with respect to the coordinate origin *O* is given by

(11) $$M_{O}=\vec{OP}\times \vec{F}=(\vec{OO_{r}}+\vec{O_{r}P})\times \vec{F}=\vec{OO_{r}}\times \vec{F}+\vec{O_{r}P}\times \vec{F},$$

where $M_{O_{r}}=\vec{O_{r}P}\times \vec{F}$; $M_{O_{r}}$ is the torque at point *P* relative to the center $O_{r}$, thus

(12) $$\vec{OO_{r}}\times \vec{F}=\left|\begin{array}{ccc} \vec{i} & \vec{j} & \vec{k}\\ h_{x} & h_{y} & 0\\ F_{x} & F_{y} & 0 \end{array}\right|=(F_{y}\cdot h_{x}-F_{x}\cdot h_{y})\vec{k}=-(M_{y}+M_{x}),$$

where *i*, *j*, and *k* are unit vectors in the *x*, *y*, and *z* directions, respectively.

(13) $$M_{O_{r}}={M_{O}+M_{x}+M_{y}=M}_{O}+F_{x}\cdot h_{y}-F_{y}\cdot h_{x},$$

where $M_{x}=F_{x}\cdot h_{y}$, $M_{y}=-F_{y}\cdot h_{x}$, $h_{x}=-L\cdot \cos\theta$, $h_{y}=-L\cdot \sin\theta$, and $\theta =\theta_{m}sin\left(2\pi ft\right)$; *F*_*x*_, *F*_*y*_, and $M_{O},$ respectively, represent the drag, lift, and torque of the cylinder monitored in the calculation, and *L* is the distance from the heaving center of the flap to the center of the cylinder.

Thus, the torque coefficient $C_{m\omega }$ in the relative heaving coordinate can be calculated as $C_{m\omega }=\frac{M_{O_{r}}}{0.5\rho c^{3}U_{\infty }^{2}}$.

### 2.3. Grid Sensitivity and Method Validation

Based on the same operating conditions as Kinsey and Dumas [9] (NACA0015, *Re* = 5 × 10^5^, *h_m_*/*c* = 1, b=1/3c, $f^{*}=fc/U_{\infty }=0.14$, and *θ_m_* = 75°), with the outside station area grid unchanged and near-wall distance $y^{+}\leq 1$, only the grid density around the airfoil is adjusted to verify the sensitivity of the original flap to grid and time-step size. As shown in Table 1, the errors caused by the grid nodes around the airfoil and the number of time steps per motion cycle are minimal. Considering the balance between computational resources and accuracy, subsequent studies will use a grid with 150 nodes around the airfoil and 2858 iteration time steps per motion cycle for further research. Meanwhile, for the subsequent analysis of the gap width, it is necessary to test the sensitivity of grid density at the gap between the airfoil and cylinder. Under fixed conditions and parameters as mentioned above, Table 2 provides the influence of different grid densities at the gap on various coefficients of the MEFW. It can be observed that the effect on the efficiency of energy harvesting by the flapping wing can be neglected once the number of grid nodes within the gap reaches 60. Therefore, for the subsequent analysis, a grid with 60 nodes within the gap will be selected for computation.

**Figure 4:**
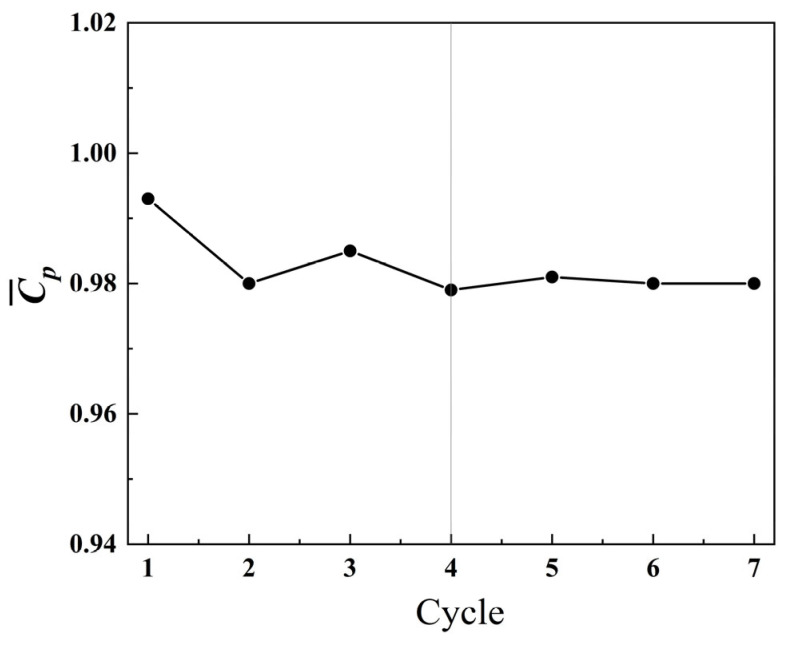
Variation in the average power coefficient ${\bar{C}}_{p}$ with the number of motion cycles.

To ensure the statistical convergence of the computational results, the variation curve of the average power coefficient with the number of motion cycles was tested, as shown in Figure 4. It can be observed that after more than four motion cycles, the fluctuation of the ${\bar{C}}_{p}$ value becomes negligible, indicating that the numerical solution has reached a periodic stable state. To ensure that subsequent computational results reach a periodic stable state, the total duration of iterations for the following computations is set to seven motion cycles, and the results of the last cycle are used for post-processing analysis.

**Figure 5:**
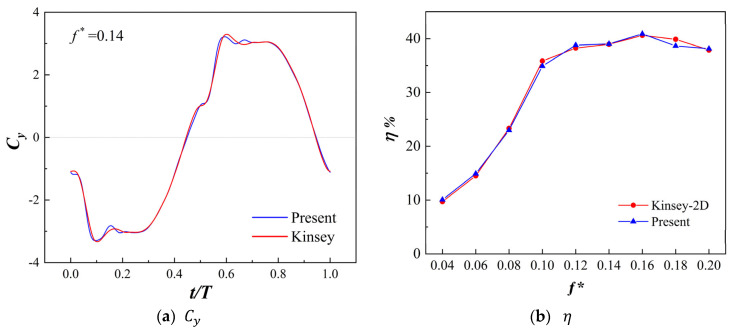
Comparison of computational results with Kinsey and Dumas’s numerical calculations [9].

To validate the accuracy of the above computational approach, the computed results were compared with the numerical results by Kinsey and Dumas [9] under the same operating conditions. Figure 5a shows the variation in the lift coefficient $C_{y}$ over one cycle as a function of time, while Figure 5b presents the variation in flapping-wing energy harvesting efficiency with different non-dimensional frequencies. It can be observed that the results obtained using the computational approach in this study closely match Kinsey’s results, indicating that the numerical method employed in this study is feasible within a certain range of accuracy.

## 3. Results

Prior investigations into fixed-wing aircraft employing the leading-edge Magnus effect have highlighted the significance of gap width and rotation ratio as pivotal parameters impacting the lift and drag characteristics of airfoils. In the context of the flapping-wing system under examination in this study, it is imperative to also consider the influence of the phase difference between the leading-edge cylinder and the flapping wing.

Using the same operating conditions and airfoil (NACA0015, *Re* = 5 × 10^5^, *h_m_*/*c* = 1, b=1/3c, *θ_m_* = 75°) as in previous research [31], it has been demonstrated that a non-dimensional frequency of $f^{*}=fc/v=$0.14 leads to higher flapping-wing energy harvesting efficiency. Therefore, subsequent investigations will focus on examining the effects of control parameters of the leading-edge Magnus rotating cylinder (gap width $a^{*}$ = a/*c* = 0.0005~0.006, rotation ratio *R* = 1~8, and phase difference $\phi_{0}\;$ = −90°~90°) on the flapping-wing energy harvesting efficiency at this frequency and under these parameters. Initially, the geometric model of the MEFW will be established by evaluating various gap width parameters denoted as $a^{*}$. Subsequently, this study will investigate the impact of different rotation speed ratios and phase differences on the energy harvesting characteristics.

**Figure 6:**
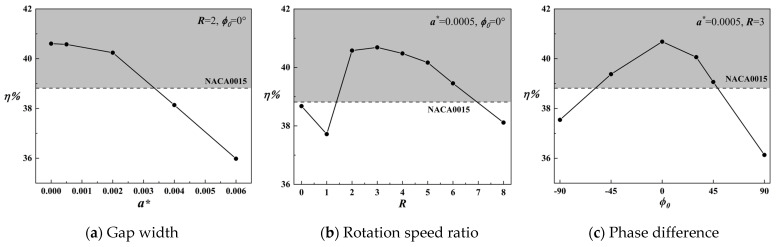
Influence of control parameter variations in the leading-edge rotating cylinder on the energy harvesting efficiency of the MEFW.

Figure 6 depicts the effects of varying control parameters on the energy harvesting efficiency of the flapping-wing device. The results demonstrate that embedding a rotating cylinder utilizing the leading-edge Magnus effect can significantly enhance the energy harvesting efficiency of the flapping-wing device, with effective control parameter ranges identified. Subsequent in-depth analyses will delve into the energy harvesting characteristics and flow field of the MEFW, aiming to investigate the individual impacts of each parameter.

### 3.1. Impact of Gap Width

**Figure 7:**
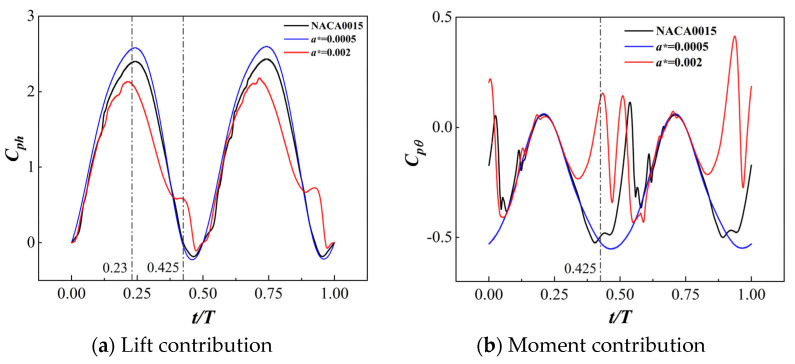
Comparison of lift coefficient contribution and moment coefficient contribution of MEFWs with different gap widths over time.

From Figure 6a, it can be observed that, while maintaining a constant rotation ratio *R* = 2 and a phase difference $\phi_{0}\;$ = 0°, the energy harvesting efficiency of the MEFW decreases with increasing gap width. Therefore, efforts should be made to minimize the width of the gap. Subsequently, considering the original NACA0015 foil as a reference, MEFWs with smaller ($a^{*}=0.0005$) and larger ($a^{*}=0.002$) gap width parameters are selected for a comparative analysis of their contributions to lift and moment forces as well as a study of the flow field. Analysis of Figure 7a reveals noticeable differences in the lift coefficient contributions of MEFWs with different gap widths around *t* = 0.23*T* during the first half-motion cycle. Similarly, MEFWs with a larger gap width ($a^{*}=0.002$) also exhibit significant differences around *t* = 0.425*T*, as evident from Figure 7b which shows the moment coefficient contributions. To investigate the impact of lift and moment coefficient contributions on energy harvesting characteristics, further comparative analysis of the flow field at the distinct time points *t* = 0.23*T* and *t* = 0.425*T* will be conducted.

**Figure 8:**
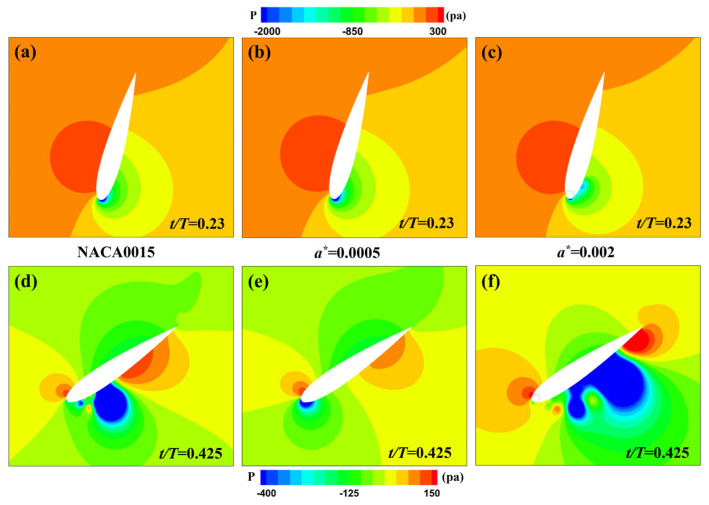
Pressure distribution around NACA0015 wing and MEFWs with different gap widths. (**a**) NACA0015 at *t* = 0.23*T*. (**b**) MEFW $\left(a^{*}=0.0005\right)$ at *t* = 0.23*T*. (**c**) MEFW $\left(a^{*}=0.002\right)$ at *t* = 0.23*T*. (**d**) NACA0015 at *t* = 0.425*T*. (**e**) MEFW $\left(a^{*}=0.0005\right)$ at *t* = 0.425*T*. (**f**) MEFW $\left(a^{*}=0.002\right)$ at *t* = 0.425*T*.

At *t* = 0.23*T* and *t* = 0.425*T*, Figure 8 shows the pressure distribution around the MEFWs with two gap width parameters ($a^{*}=0.0005$ and $a^{*}=0.002$) and the NACA0015 foil. It can be observed that at *t* = 0.23*T*, the pressure distribution on the pressure side of the wings is similar among the three types of wings, while the size of the low-pressure region near the leading edge on the suction side of the airfoil follows the order of $a^{*}$ = 0.0005 > original airfoil > $a^{*}$ = 0.002, consistent with the lift coefficient contributions at this time point shown in Figure 7a. At *t* = 0.425*T*, with increasing gap width, the high-pressure region at the leading edge of the wing gradually enlarges, the low-pressure region below the cylinder decreases and disappears, and a gradually enlarging low-pressure region appears on the lower surface of the wing with increasing gap width. Additionally, the high-pressure region at the trailing edge also increases with the increase in gap width, similar to the presence of a low-pressure region on the lower surface of the NACA0015 wing. Considering the downward motion of the wing at this time, the low-pressure region below the wing is conducive to the wing’s motion. Therefore, the NACA0015 wing and the MEFW with a larger gap width exhibit higher moment coefficient contributions after this time point.

**Figure 9:**
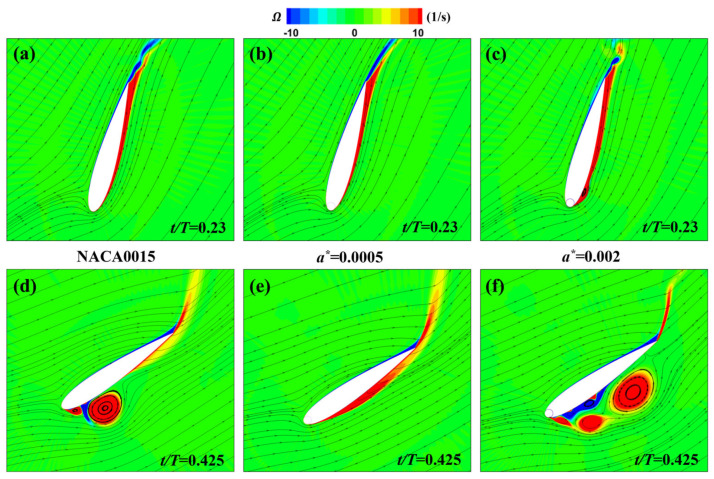
Comparison of vorticity and streamlines around the wings with different gap widths. (**a**) NACA0015 at *t* = 0.23*T*. (**b**) MEFW $\left(a^{*}=0.0005\right)$ at *t* = 0.23*T*. (**c**) MEFW $\left(a^{*}=0.002\right)$ at *t* = 0.23*T*. (**d**) NACA0015 at *t* = 0.425*T*. (**e**) MEFW $\left(a^{*}=0.0005\right)$ at *t* = 0.425*T*. (**f**) MEFW $\left(a^{*}=0.002\right)$ at *t* = 0.425*T*.

At *t* = 0.23*T* and *t* = 0.425*T*, Figure 9 shows the vorticity and streamline distributions around the MEFWs with two gap width parameters ($a^{*}$ = 0.0005 and $a^{*}$ = 0.002) and the NACA0015 wing. At *t* = 0.23*T*, the MEFW with a gap width of $a^{*}$ = 0.002 exhibits smaller leading-edge vortex shedding on the lower surface of the wing, thereby reducing the lift and its contribution to the power harvesting (see Figure 7a). At *t* = 0.425*T*, a large separation vortex forms on the lower surface of the traditional NACA0015 wing, corresponding to the negative pressure distribution in Figure 8d. In contrast, no separation vortex is observed on the lower surface of the MEFW with $a^{*}$ = 0.0005, indicating that the rotating cylinder significantly inhibits the formation of separation vortices. For the MEFW with $a^{*}$ = 0.002, it can be observed that the shed vortex has moved along the lower surface of the wing towards the middle and rear sections, consistent with the distribution of the low-pressure region in Figure 8f. At this time, the continuous shed vortices generated beneath the $a^{*}$ = 0.002 MEFW contribute to increasing the lift and its power extraction (see Figure 7a). Additionally, as the wing needs to rotate clockwise around the pitching center to return to the horizontal position, the shed vortices moving along the lower surface of the wing reach behind the pitching center. The negative pressure distribution at the vortex center produces a favorable torque for the wing’s pitching motion, thereby increasing the contribution of the pitching moment to the power coefficient (see Figure 7b).

**Figure 10:**
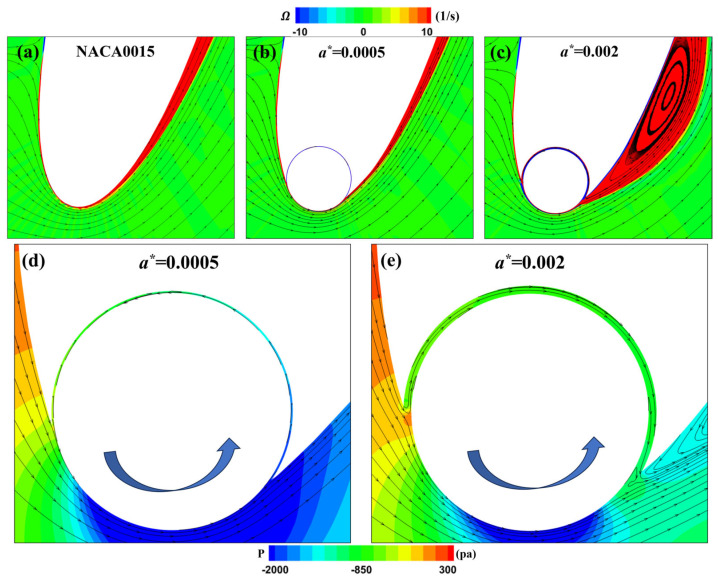
Comparison of vorticity around the leading-edge cylinder and local pressure streamlines between NACA0015 wing and MEFWs. (**a**) Local vorticity and streamlines around NACA0015 at *t* = 0.23*T*. (**b**) Local vorticity and streamlines around MEFW $\left(a^{*}=0.0005\right)$ at *t* = 0.23*T*. (**c**) Local vorticity and streamlines around MEFW $\left(a^{*}=0.002\right)$ at *t* = 0.23*T*. (**d**) Local pressure and streamlines around the leading-edge cylinder of MEFW $\left(a^{*}=0.0005\right)$ at *t* = 0.23*T*. (**e**) Local pressure and streamlines around the leading-edge cylinder of MEFW $\left(a^{*}=0.002\right)$ at *t* = 0.23*T*. **Arrow:** Rotation direction of the leading-edge cylinder.

Figure 10 shows the vorticity and velocity distributions around the leading-edge cylinder of the MEFWs with different gap width parameters ($a^{*}$ = 0.0005 and $a^{*}$ = 0.002) and the NACA0015 wing at *t* = 0.23*T*. It can be observed that the flow field differences between the MEFW with a smaller gap width ($a^{*}$ = 0.0005) and the NACA0015 wing are not significant. However, due to the flow induced by the rotation of the cylinder being in the same direction as the mainstream flow, it increases the lift and its contribution to the power coefficient without causing separation (see Figure 7a). On the other hand, the MEFW with a larger gap width ($a^{*}$ = 0.002) generates a leading-edge vortex, mainly because the pressure difference on both sides of the airfoil’s leading edge induces a flow opposite to the mainstream flow within the wider gap, triggering the formation of a separation vortex at the foil’s leading edge.

**Figure 11:**
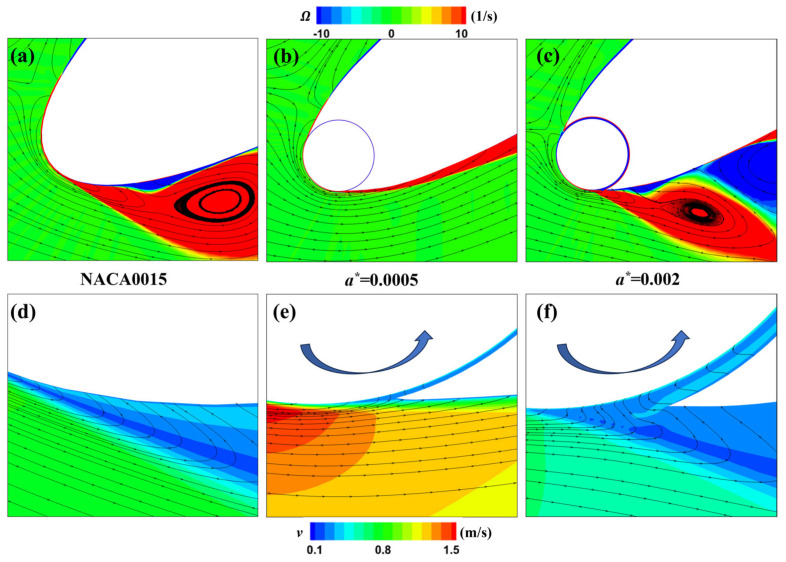
Comparison of vorticity and velocity around the leading-edge cylinder between NACA0015 wing and MEFWs. (**a**) Local vorticity and streamlines around NACA0015 at *t* = 0.425*T*. (**b**) Local vorticity and streamlines around MEFW ($a^{*}=0.0005$) at *t* = 0.425*T*. (**c**) Local vorticity and streamlines around MEFW ($a^{*}=0.002$) at *t* = 0.425*T*. (**d**) Local velocity and streamlines below the leading-edge of NACA0015 at *t* = 0.425*T*. (**e**) Local velocity and streamlines below the leading-edge cylinder of MEFW ($a^{*}=0.0005$) at *t* = 0.425*T*. (**f**) Local velocity and streamlines below the leading-edge cylinder of MEFW ($a^{*}=0.002$) at *t* = 0.425*T*. **Arrow:** Rotation direction of the leading-edge cylinder.

Figure 11 illustrates the vorticity and velocity distributions around the leading-edge cylinder of the MEFWs with different gap width parameters ($a^{*}$ = 0.0005 and $a^{*}$ = 0.002) at *t*/*T* = 0.425. For the NACA0015 wing, the high angle of attack of the flow around the foil’s leading edge leads directly to the formation of separation vortices and low-speed recirculation below the foil’s leading edge. For the MEFW with $a^{*}$ = 0.0005, as shown in the streamline distributions in Figure 11b,e, the counterclockwise rotation of the cylinder increases the flow velocity around the foil’s leading edge, thereby reducing the angle of attack of the flow around the foil’s leading edge and effectively suppressing flow separation. However, for the MEFW with $a^{*}$ = 0.002, the widening of the gap width causes fluid from the pressure side of the foil to flow to the suction side through the gap (see Figure 10e). At this point, the flow velocity at the gap exit is opposite to the mainstream flow direction, leading to the reformation of separation vortices. Compared to the traditional NACA0015 wing, the combined effect of the leading-edge rotating cylinder and the leakage flow from the gap changes the timing and state of separation vortex shedding, resulting in lift and pitching moment conducive to energy harvesting. This is also why the MEFW with $a^{*}$= 0.002 can improve the energy harvesting efficiency. Overall, it is advisable to reduce the gap width to minimize the boundary layer separation caused by leakage flow. Additionally, future efforts could explore the placement of isolating pillars within the gap to mitigate the influence of gap leakage on the boundary layer flow on the suction side.

### 3.2. Impact of Rotation Speed Ratio

**Figure 12:**
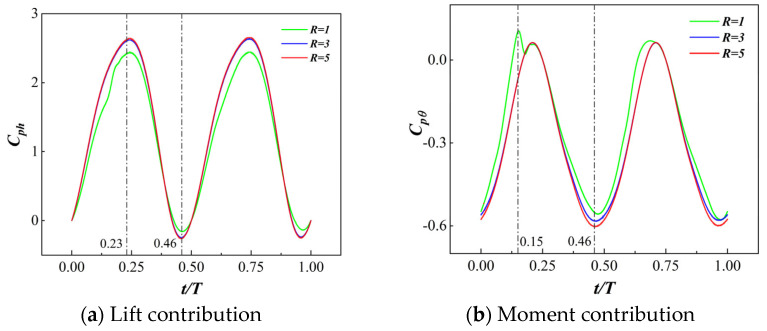
Comparison of power and moment coefficients’ evolution over time for different speed ratios.

Maintaining a gap width of $a^{*}$ = 0.0005 and a phase difference of $\phi_{0}$ = 0°, we selected MEFW configurations with three discrete rotational speed ratios (*R* = 1, 3, 5) in Figure 6b as the research subjects. Figure 12 illustrates the variations in lift coefficient and moment coefficient for the MEFWs at different rotation speed ratios. It is evident that during the first half-cycle, there are significant differences in the lift coefficient at *t*/*T* = 0.23 and 0.46 among the different speed ratios, while the moment coefficient for the *R* = 1 MEFW differs from the others at *t*/*T* = 0.15. Further in-depth analysis will be conducted on the flow field of MEFWs with different speed ratios at these time points.

**Figure 13:**
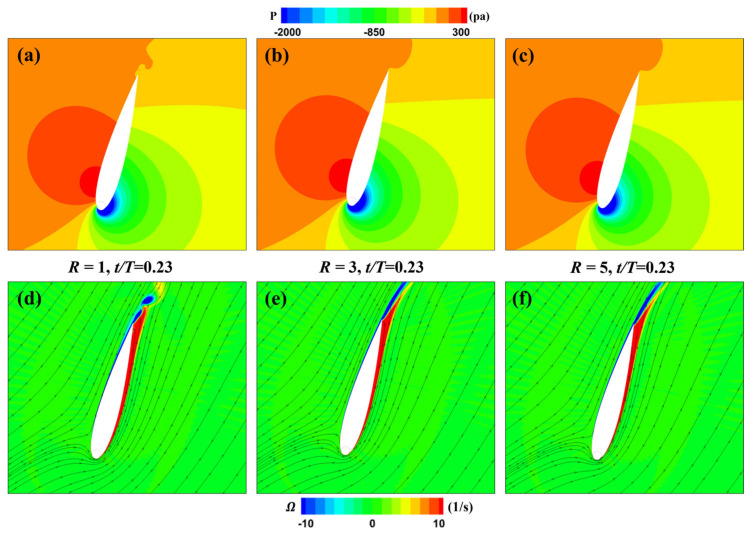
Distribution of pressure and vorticity for MEFWs with different rotation speed ratios. (**a**) Pressure around MEFW (*R* = 1) at *t* = 0.23*T*. (**b**) Pressure around MEFW (*R* = 3) at *t* = 0.23*T*. (**c**) Pressure around MEFW (*R* = 5) at *t* = 0.23*T*. (**d**) Vorticity and streamlines around MEFW (*R* = 1) at *t* = 0.23*T*. (**e**) Vorticity and streamlines around MEFW (*R* = 3) at *t* = 0.23*T*. (**f**) Vorticity and streamlines around MEFW (*R* = 5) at *t* = 0.23*T*.

Figure 13 depicts the pressure and vorticity distributions around the MEFWs at *t* = 0.23*T* for three different rotation speed ratios (*R* = 1, 3, 5). It can be observed that the pressure distributions among the three cases are quite similar. However, the low-pressure region on the suction side proximal to the leading edge of the MEFW with *R* = 1 is notably diminished in comparison to the MEFWs with *R* = 3 and *R* = 5. Given the current motion of the wing, the low-pressure region beneath it facilitates its movement, consequently leading to a reduced lift coefficient for the MEFW with *R* = 1 (see Figure 12a). No significant differences are observed from the vorticity distributions.

**Figure 14:**
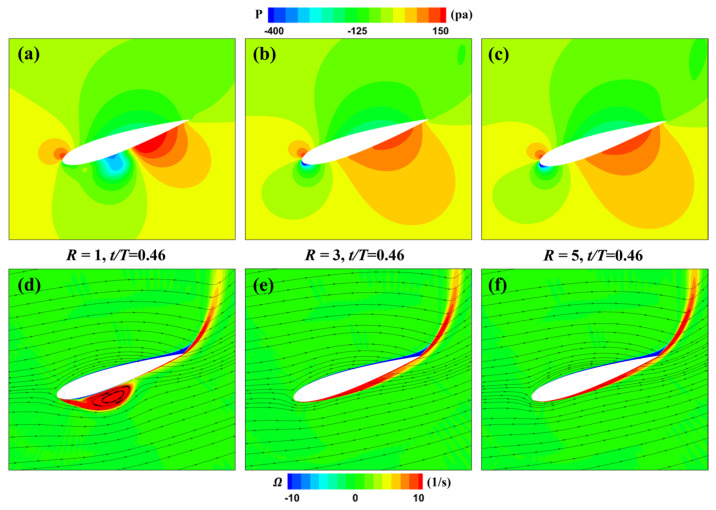
Distribution of pressure and vorticity for MEFWs with different rotation speed ratios. (**a**) Pressure around MEFW (*R* = 1) at *t* = 0.46*T*. (**b**) Pressure around MEFW (*R* = 3) at *t* = 0.46*T*. (**c**) Pressure around MEFW (*R* = 5) at *t* = 0.46*T*. (**d**) Vorticity and streamlines around MEFW (*R* = 1) at *t* = 0.46*T*. (**e**) Vorticity and streamlines around MEFW (*R* = 3) at *t* = 0.46*T*. (**f**) Vorticity and streamlines around MEFW (*R* = 5) at *t* = 0.46*T*.

Figure 14 illustrates the pressure and vorticity distributions around the MEFWs at *t* = 0.46*T* for three different rotating speed ratios (*R* = 1, 3, 5). It can be observed that for the MEFW with *R* = 1, a prominent leading-edge vortex appears on the lower surface, reducing the near-wall vorticity at the rear of the wing. As shown in Figure 14a, a low-pressure region forms in the middle section of the wing’s lower surface. At this moment, the wing is rotating clockwise to return to the horizontal position, and this pressure distribution reduces the ability of the wing to generate negative lift-induced torque (refer to Figure 12b). Additionally, the negative pressure induced by the leading-edge vortex also decreases the ability of the wing to generate negative lift-induced torque (refer to Figure 12a). The lift-induced powers of the MEFWs with *R* = 3 and *R* = 5 are similar, but the pitching moment-induced negative power of the MEFW with *R* = 3 is smaller than that of *R* = 5, thus the energy acquisition capability of the MEFW with *R* = 3 is higher.

**Figure 15:**
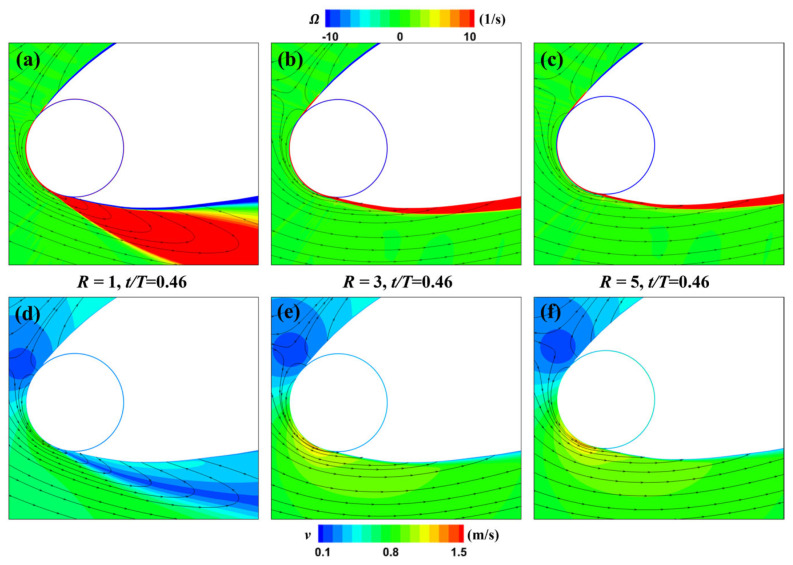
Distribution of local vorticity and velocity for MEFWs with different rotation speed ratios. (**a**) Local vorticity and streamlines around MEFW (*R* = 1) at *t* = 0.46*T*. (**b**) Local vorticity and streamlines around MEFW (*R* = 3) at *t* = 0.46*T*. (**c**) Local vorticity and streamlines around MEFW (*R* = 5) at *t* = 0.46*T*. (**d**) Local velocity and streamlines around MEFW (*R* = 1) at *t* = 0.46*T*. (**e**) Local velocity and streamlines around MEFW (*R* = 3) at *t* = 0.46*T*. (**f**) Local velocity and streamlines around MEFW (*R* = 5) at *t* = 0.46*T*.

To better elucidate the influence of different rotation speed ratios on the flow field at this moment, we have locally magnified and displayed the vorticity and velocity fields around the leading-edge cylinder for the three different speed ratios of the MEFWs, as depicted in Figure 15. It is evident that due to the smaller rotation speed ratio of *R* = 1, the slowly rotating cylinder is insufficient to control the formation of separation vortices beneath the leading edge of the wing, resulting in a large low-velocity region in this area. In contrast, the corresponding regions for higher rotation speed ratios effectively control the generation of separation vortices and backflow induced by the rotating cylinder.

**Figure 16:**
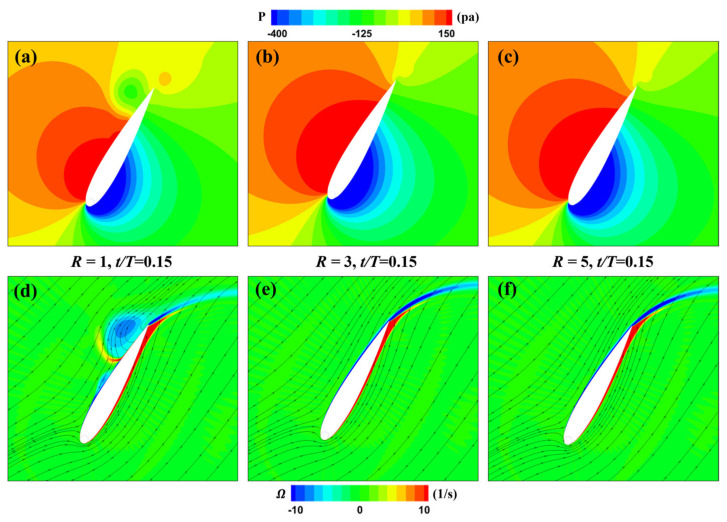
Pressure and vorticity distribution of MEFWs with different rotation speed ratios. (**a**) Pressure around MEFW (*R* = 1) at *t* = 0.15*T*. (**b**) Pressure around MEFW (*R* = 3) at *t* = 0.15*T*. (**c**) Pressure around MEFW (*R* = 5) at *t* = 0.15*T*. (**d**) Vorticity and streamlines around MEFW (*R* = 1) at *t* = 0.15*T*. (**e**) Vorticity and streamlines around MEFW (*R* = 3) at *t* = 0.15*T*. (**f**) Vorticity and streamlines around MEFW (*R* = 5) at *t* = 0.15*T*.

Figure 16 shows the pressure and vorticity distributions around the MEFWs at *t* = 0.15*T* for the three rotation speed ratios (*R* = 1, 3, 5). It can be observed that the flow fields for the *R* = 3 and 5 MEFWs are almost identical, while for the *R* = 1 MEFW, there is a separation vortex near the trailing edge of its pressure surface, which is generated by the separation of the leading-edge vortex formed in the previous instant, like the situation at *t* = 0.46*T*. This vortex also creates a low-pressure region in the corresponding area (see Figure 16a), thereby reducing the lift and its contribution to the power extraction. However, since the wing is rotating counterclockwise at this instant, the low-pressure region caused by this vortex favors the generation of counterclockwise rotation torque, hence the higher torque contribution to power extraction for the *R* = 1 MEFW (see Figure 12b).

In summary, when the rotation speed ratio is relatively low, the rotating cylinder is insufficient to control the separation vortex at the wing’s leading edge. As the speed ratio increases, the rotating cylinder suppresses the generation of a separation vortex at the wing’s leading edge. However, for the MEFWs with *R* = 3 and 5, the lift contributions are essentially the same, while the pitching moment contributions begin to decrease as the rotation speed increases, and the power consumed by cylinder rotation starts to increase (see Table 3). Therefore, under the conditions studied in this work, a rotation speed ratio of *R* = 3 is recommended to optimize the energy harvesting efficiency.

### 3.3. Impact of Phase Difference

With the optimal values of the gap width and speed ratio determined ($a^{*}$ = 0.0005, *R* = 3), four representative phase difference parameters ($\phi_{0}\;$= −90°, 0°, 45°, 90°) were selected based on Figure 6c to further investigate the effect of phase differences on the energy harvesting efficiency of the MEFW.

**Figure 17:**
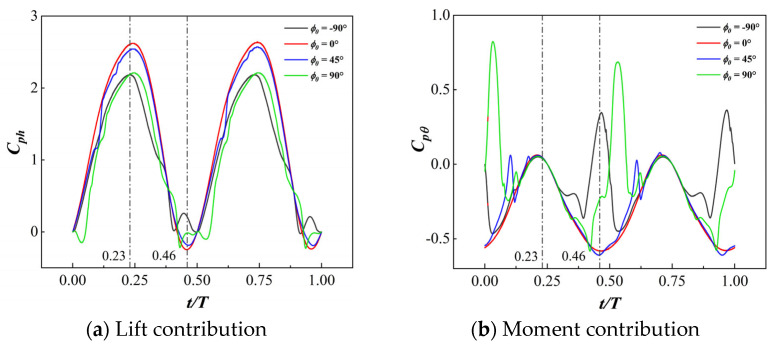
Comparison of lift and moment coefficients over time for MEFWs with different phase differences.

Figure 17 compares the lift coefficient and pitching moment coefficient of the MEFW at different phase differences. It can be observed that at *t* = 0.23*T*, there is significant variation in the lift coefficient among MEFWs with different phase differences, while there is almost no difference in the pitching moment coefficients. However, at *t* = 0.46*T*, both the lift coefficient and pitching moment coefficient show differences among MEFWs with different phase differences.

**Figure 18:**
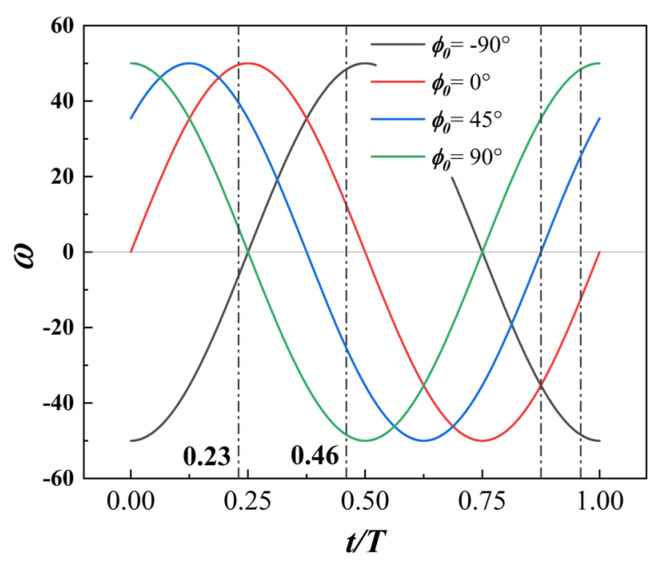
Variation in the rotational speed of the leading-edge cylinder for different phase differences $\phi_{0}$.

Based on the definition of the cylinder phase difference $\phi_{0}$ provided earlier, Figure 18 illustrates the variation in the cylinder rotation speed throughout the entire motion cycle of the MEFW.

**Figure 19:**
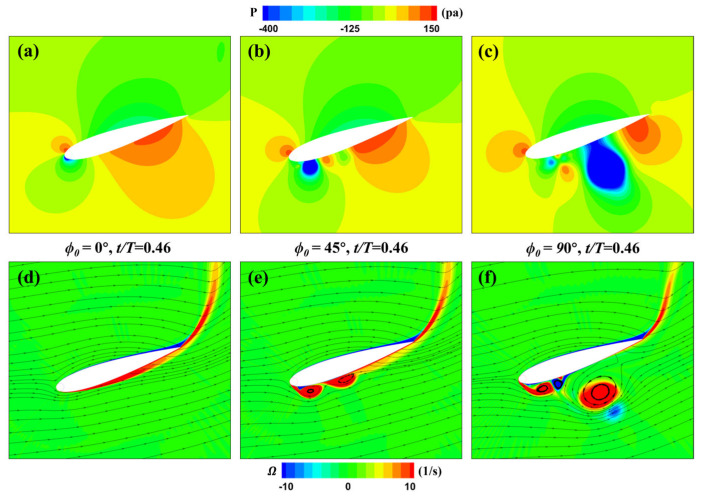
Influence of phase difference parameters on the MEFWs. (**a**) Pressure around MEFW ($\phi_{0}\;$= 0°) at *t* = 0.46*T*. (**b**) Pressure around MEFW ($\phi_{0}\;$ = 45°) at *t* = 0.46*T*. (**c**) Pressure around MEFW ($\phi_{0}\;$ = 90°) at *t* = 0.46*T*. (**d**) Vorticity and streamlines around MEFW ($\phi_{0}\;$ = 0°) at *t* = 0.46*T*. (**e**) Vorticity and streamlines around MEFW ($\phi_{0}\;$ = 45°) at *t* = 0.46*T*. (**f**) Vorticity and streamlines around MEFW ($\phi_{0}\;$ = 90°) at *t* = 0.46*T*.

At fixed instants of *t*/*T* = 0.23 and 0.46, different phase differences $\phi_{0}$ affect the current rotation speed of the cylinder. Figure 19 provides a comparison of the flow field distribution of three MEFWs at *t* = 0.46*T*, where there is a significant difference in rotational speed. It can be observed that the vorticity distribution on the lower surface of the wing is relatively uniform for $\phi_{0}\;$= 0°, while $\phi_{0}\;$= 45° and 90° result in the formation of two distinct vortices on the lower surface of the wing. Additionally, for $\phi_{0}\;$ = 90°, the trailing-edge vortex has moved far away from the wing surface, leading to the formation of low-pressure regions on the lower surface of the wing. As analyzed earlier, these vortices contribute positively to the lift and pitching moment of the flapping wing in this instance. Consequently, in Figure 17, it can be observed that at this moment, the lift coefficient and moment coefficient for the wing with $\phi_{0}\;$ = 90° are higher than those for $\phi_{0}\;$ = 0° and 45°.

**Figure 20:**
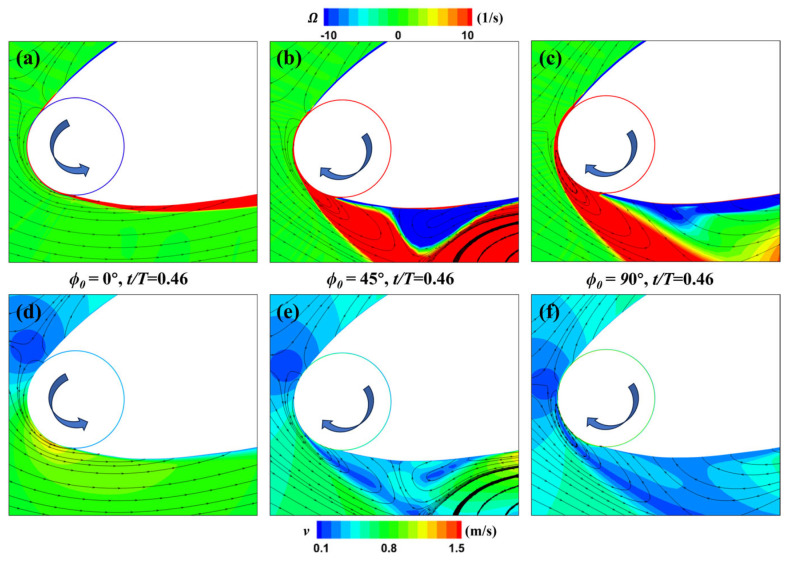
Influence of phase difference parameters on the MEFWs. (**a**) Local vorticity and streamlines around MEFW ($\phi_{0}\;$ = 0°) at *t* = 0.46*T*. (**b**) Local vorticity and streamlines around MEFW ($\phi_{0}\;$ = 45°) at *t* = 0.46*T*. (**c**) Local vorticity and streamlines around MEFW ($\phi_{0}\;$ = 90°) at *t* = 0.46*T*. (**d**) Local velocity and streamlines around MEFW ($\phi_{0}\;$ = 0°) at *t* = 0.46*T*. (**e**) Local velocity and streamlines around MEFW ($\phi_{0}\;$ = 45°) at *t* = 0.46*T*. (**f**) Local velocity and streamlines around MEFW ($\phi_{0}\;$ = 90°) at *t* = 0.46*T*. **Arrow:** Rotation direction of the leading-edge cylinder.

To analyze the cause of the flow field differences in this instance, the flow field in the leading-edge region is magnified, as shown in Figure 20. According to the different rotation speed distributions for various phase differences shown in Figure 18, at *t* = 0.46*T*, the rotational speed of the leading-edge cylinder for $\phi_{0}\;$ = 0° is relatively low, while for $\phi_{0\;}$= 45° and 90°, the rotational speed increases progressively, and the rotation direction is opposite to that of $\phi_{0}\;$ = 0°. Consequently, the flow velocity beneath the leading-edge cylinder for $\phi_{0}\;$= 0° remains uniform without generating any disturbances; therefore, no formation of leading-edge vortices occurs. In contrast, for $\phi_{0}\;$ = 45° and 90°, due to the rotation direction being opposite to the incoming flow direction, significant separation leading-edge vortices and low-speed regions are formed in this area. Additionally, since the rotational speed for $\phi_{0}\;$ = 90° is higher than that for $\phi_{0}\;$= 45°, the increased angle of attack forces the boundary layer separation closer to the wing leading edge. This is also the primary reason why the leading-edge vortices in Figure 19c quickly detach from the wing.

**Figure 21:**
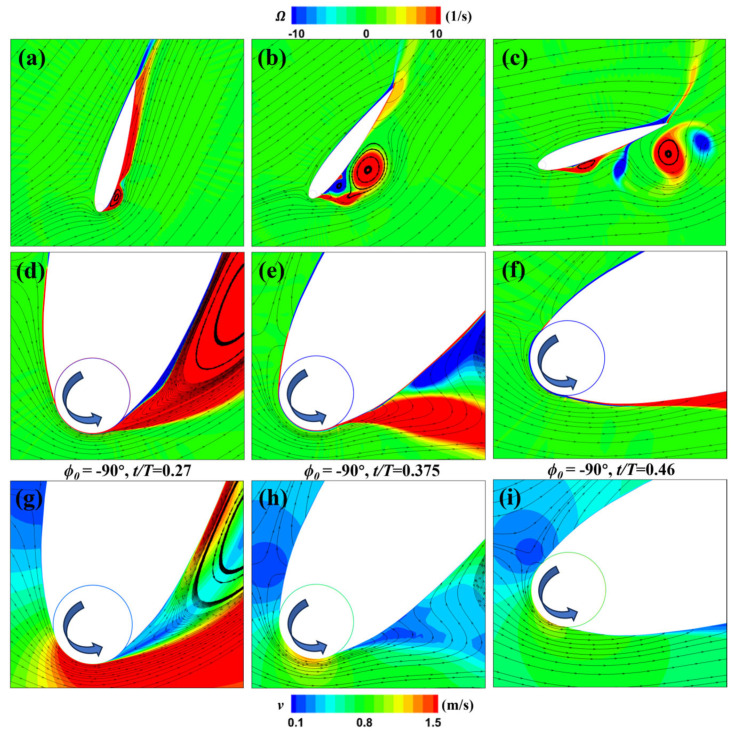
Vorticity and velocity distributions of the MEFW. (**a**) Vorticity and streamlines around MEFW ($\phi_{0}$ = −90°) at *t* = 0.27*T*. (**b**) Vorticity and streamlines around MEFW ($\phi_{0}$ = −90°) at *t* = 0.375*T*. (**c**) Vorticity and streamlines around MEFW ($\phi_{0}$ = −90°) at *t* = 0.46*T*. (**d**) Local vorticity and streamlines around MEFW ($\phi_{0}\;$= −90°) at *t* = 0.27*T*. (**e**) Local vorticity and streamlines around MEFW ($\phi_{0}$ = −90°) at *t* = 0.375*T*. (**f**) Local vorticity and streamlines around MEFW ($\phi_{0}\;$= −90°) at *t* = 0.46*T*. (**g**) Local velocity and streamlines around MEFW ($\phi_{0}\;$= −90°) at *t* = 0.27*T*. (**h**) Local velocity and streamlines around MEFW ($\phi_{0}$ = −90°) at *t* = 0.375*T*. (**i**) Local velocity and streamlines around MEFW ($\phi_{0}\;$= −90°) at *t* = 0.46*T*. **Arrow:** Rotation direction of the leading-edge cylinder.

For the MEFW with $\phi_{0}\;$ = −90°, Figure 17 shows that at *t* = 0.46*T*, both the lift and pitch moment coefficients exhibit a local peak. To investigate the reasons behind this phenomenon, Figure 21 presents the evolution of the flow field of the MEFW with $\phi_{0}$ = −90° over time. It can be observed that at *t* = 0.27*T*, the change in direction of the wing’s leading-edge cylinder results in lower flow velocity beneath the leading edge, inducing the formation of a leading-edge vortex. At *t* = 0.375*T*, although the rotation direction of the cylinder is consistent with the flow velocity direction around the wing’s leading edge, the lower rotational speed weakens the control over the leading-edge separation vortex at this angle of attack, hence the presence of the leading-edge separation vortex beneath the wing. By *t* = 0.46*T*, although the reduction in leading-edge rotation and the decrease in the wing’s angle of attack suppress the formation of the leading-edge separation vortex, the previously separated leading edge has moved along the wing surface toward the trailing edge, resulting in a local low-pressure area at the vortex core position. The generated low-pressure area increases the lift and pitch moment, thus enhancing the positive lift and pitch moment coefficients of the MEFW with $\phi_{0}\;$ = −90° around this time.

In summary, compared to the MEFW without a phase difference ($\phi_{0}\;$ = 0°), the MEFW with a phase difference benefits from a velocity field induced by the rotating cylinder at its leading edge, which perfectly matches the incoming flow velocity field around the wing’s leading edge. Consequently, this generates Magnus force acting in the same direction as the flapping motion, thereby enhancing the overall power by lift force (see Figure 17a).

## 4. Conclusions

Based on the Magnus principle, this paper presents a novel flapping energy harvesting device that incorporates a rotating cylinder embedded at the leading edge of the foil. Employing a transient numerical simulation method based on sliding mesh technology within a relative heaving-motion reference frame, alongside an analysis of vortical structures and pressure distributions surrounding the flapping wing, this study delves into the principle of enhancing flapping energy harvesting characteristics with a Magnus rotating cylinder. The effects of leading-edge cylinder gap width, rotation speed ratio, and phase difference on the energy harvesting characteristics of the flapping wing are meticulously scrutinized. The primary conclusions are outlined as follows:

1. Embedding a rotating cylinder at the wing’s leading edge can enhance the energy harvesting capability of the flapping device. This is because the leading-edge rotating cylinder can contribute lift force itself while also suppressing the generation of leading-edge vortices, thereby enhancing the lift force effectiveness of the flapping wing.

2. The control parameters of the leading-edge rotating cylinder greatly influence the energy harvesting efficiency of the flapping device. A smaller gap width reduces the impact of reverse leakage flow at the gap outlet on boundary layer separation, thereby suppressing the formation of separation vortices and increasing the energy harvesting efficiency of the flapping wing as the gap width decreases. The energy harvesting efficiency of the Magnus flapping wing initially increases and then decreases with an increase in rotation speed ratio, so the choice of rotation speed ratio must balance the enhancement in lift force due to the suppression of separation vortices with the power consumption required for driving cylinder rotation. The phase difference between the motion of the rotating cylinder and the flapping wing directly affects the magnitude and angle of attack of the resultant flow around the wing’s leading edge, as well as the degree of matching between the Magnus force and the flapping-wing motion. Under the operating conditions examined in this study, the optimized parameters for achieving high energy harvesting efficiency are a gap width of $a^{*}$ = 0.0005, a speed ratio of *R* = 3, and a phase difference of $\phi_{0}\;$ = 0°.

## Figures and Tables

**Table 1:** Numerical results comparison for different grid nodes and time steps (ts).

Numbers	Nodes	ts/Cycle	y+	${\bar{C}}_{y}$	$C_{p}$	η
90,135	100	2858	1.0	2.779	0.974	0.381
18,105	150	2858	0.9	2.845	0.980	0.384
18,105	150	1429	0.9	2.834	0.978	0.383
18,105	150	4287	0.8	2.856	0.982	0.385
271,008	200	2858	0.7	2.867	0.984	0.386

**Table 2:** Numerical results comparison for different grid nodes in gap.

Nodes in Gap	${\bar{C}}_{y}$	$C_{p}$	η
20	2.587	0.958	0.372
40	2.842	0.976	0.381
60	2.845	0.980	0.384
80	2.844	0.981	0.384

**Table 3:** Energy harvesting coefficients of MEFWs with different rotation speed ratios.

*R*	${\bar{C}}_{p\omega }$	${\bar{C}}_{ph}$	${\bar{C}}_{p\theta }$	η (%)
1	−0.000964	1.21	−0.25	37.719
3	−0.00414	1.34	−0.30	40.685
5	−0.018	1.35	−0.31	40.166

## Data Availability

The original contributions presented in this study are included in this article; further inquiries can be directed to the corresponding author.
